# Refractory Disseminated Intravascular Coagulation Caused by Endovascular Invasion of Squamous Cell Carcinoma of the Lung: A Case Report

**DOI:** 10.7759/cureus.84243

**Published:** 2025-05-16

**Authors:** Yutaka Tsukamoto, Masataka Umeda, Takashi Sugimoto, Yuki Matsuoka, Takeharu Kato, Masahiro Nakashima, Koya Ariyoshi, Takahiro Maeda

**Affiliations:** 1 Department of Clinical Medicine, Institute of Tropical Medicine, Nagasaki University, Nagasaki, JPN; 2 Department of Immunology and Rheumatology, Nagasaki University Graduate School of Biomedical Sciences, Nagasaki, JPN; 3 Department of General Medicine, Nagasaki University Graduate School of Biomedical Sciences, Nagasaki, JPN; 4 Department of Pathology, Nagasaki University Hospital, Nagasaki, JPN; 5 Department of Hematology, Nagasaki University Hospital, Nagasaki, JPN

**Keywords:** disseminated intravascular coagulation, endothelium damage, endovascular invasion, lung cancer, squamous cell carcinoma

## Abstract

Disseminated intravascular coagulation (DIC) is a systemic intravascular activation of coagulation caused by various underlying conditions. Severe DIC cases could be fatal. We present a 78-year-old Japanese man who developed uncontrollable DIC, finally diagnosed as lung cancer and intravascular invasion along the endothelium of the pulmonary artery, massive pulmonary embolism, and tumor embolism in autopsy. Leucocytes, platelets, and cancer cells play roles in uncontrollable DIC by assisting the attachment of cancer cells to endothelia. Endothelium damage enhances coagulation and suppresses fibrinolysis. This is the first case of uncontrollable DIC caused by intravascular invasion of lung cancer along the endothelium.

## Introduction

Disseminated intravascular coagulation (DIC) is a systemic abnormal activation of the coagulation and thrombolysis system, resulting in both thrombosis and hemorrhage because of consumption of platelets and coagulation factors. Underlying causes include infection, malignancies, obstetric complications, pancreatitis, trauma, and vascular disorders (e.g., aortic aneurysms) [1,2]. Damage to the endothelium activates tissue factor release in blood, and interaction between cancer cells and platelets triggers platelet activation [1,3].

Several articles on intravascular invasion of lung cancer have been published [4-9]. It is not a common clinical situation, but it can cause significant damage to pulmonary vessels, which leads to DIC.

We present a patient with DIC from an unknown cause, and autopsy revealed lung cancer (synchronous squamous cell carcinoma and adenocarcinoma), pulmonary artery invasion of squamous cell carcinoma, pulmonary embolism, and tumor embolism. To our knowledge, this is the first case of uncontrollable DIC caused by tumor invasion into the pulmonary artery.

## Case presentation

**Figure 1:**
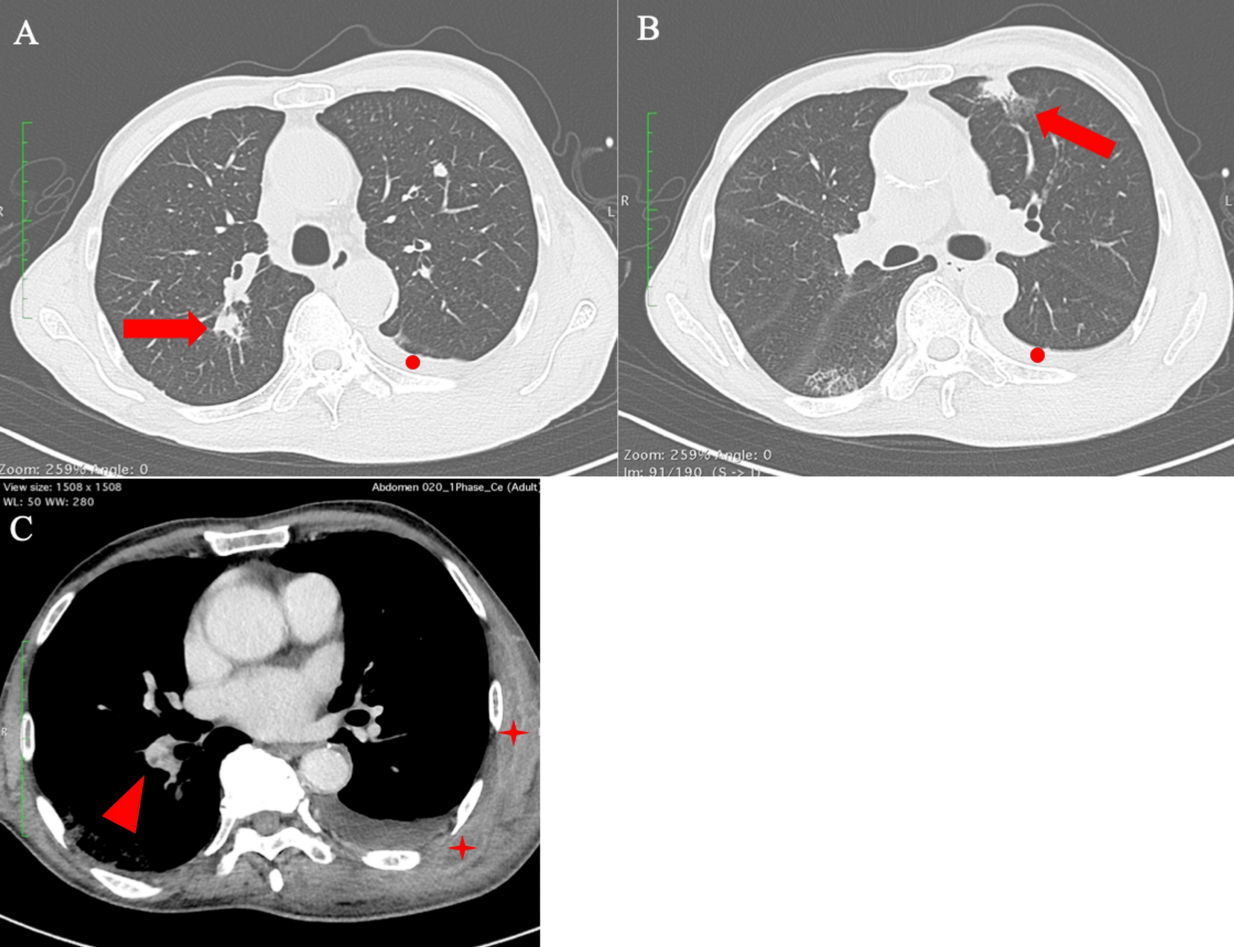
Chest contrast-enhanced computed tomography A and B: Pleural effusion in the left lung (red circles) and lung nodules in both lungs (red arrows). C: Pulmonary embolism in the right pulmonary artery (red arrowhead) and intramuscular hematomas in the left back (red stars).

A 78-year-old Japanese man was referred to our hospital for evaluation of the cause of his chronic DIC. He was a victim of the atomic bomb when he was three years old. He had a smoking history of 100 pack-years. Fourteen months before admission, he was diagnosed with atrial fibrillation, and rivaroxaban was prescribed. Four months prior to admission, he experienced difficulty stopping bleeding when he had his teeth pulled out. He also had systemic subcutaneous hemorrhage and hematemesis. One month before admission, rivaroxaban was suspended because of his bleeding tendency, and gastroscopy revealed esophageal cancer (carcinoma in situ (Tis)). Chest computed tomography (CT) scan showed left pleural effusion, three nodules in the left upper lobe, and one nodule in the right upper lobe, and the one in the left lung was likely to be lung cancer (Figure 1).

Laboratory investigation was consistent with DIC, but the etiology was not clear. The left lung nodule could be malignancy, but the maximum diameter was 40 mm, and there was no radiologically suspected metastatic region. This is why the lung nodule was not considered a cause of DIC. This patient was therefore transferred to our hospital for further investigation.

**Figure 2:**
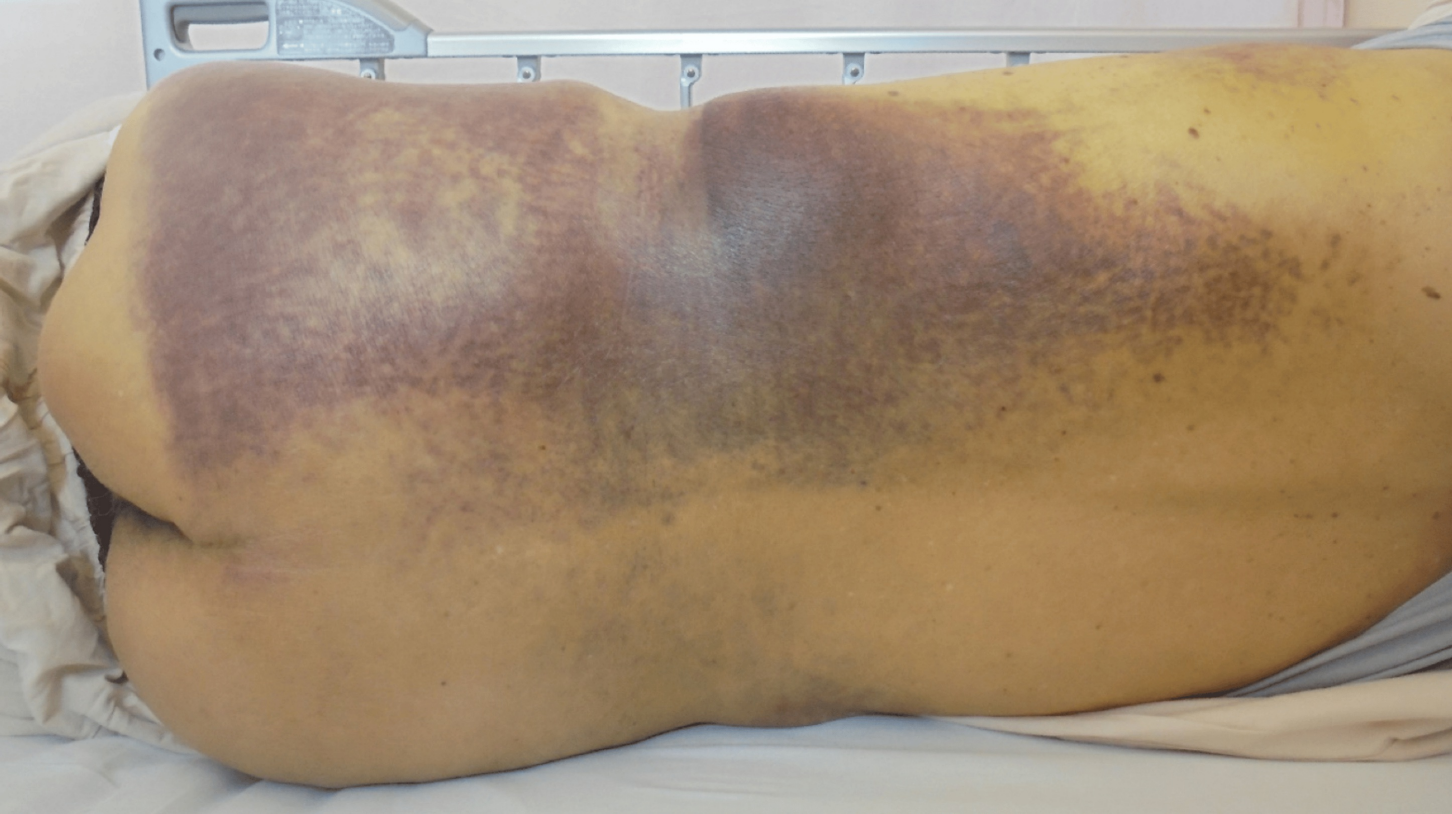
Left back swelling and subcutaneous hemorrhage

On admission to our hospital, he did not look sick, and vital signs were stable: blood pressure, 126/63 mmHg; heart rate, 94/minute; percutaneous oxygen saturation (SpO_2_), 99% (room air); and Glasgow Coma Scale, E4V5M6. His height and weight were 167 cm and 49 kg, respectively. His physical examination was remarkable for pale conjunctiva, left back swelling, and subcutaneous hemorrhage (Figure 2).

He had no mucosal bleeding, peripheral petechiae, or neurological deficit.

The initial laboratory investigation showed significant coagulation abnormality: prothrombin time (PT), international normalized ratio (INR), 1.31; activated partial thromboplastin time (APTT), 40.3 seconds; fibrinogen, 96 mg/dL (reference range: 200-400 mg/dL); antithrombin activity, 99% (reference range: 80%-130%); fibrin degradation product (FDP), 98.7 μg/mL (normal value: <5 μg/mL); and D-dimer (DD), 33.2 μg/mL (normal value: <1.0 μg/mL). Platelet count was normal at 274×10^9^/L. The following findings were also abnormal: white blood cell (WBC) count, 11.6×10^9^/L; neutrophils, 9.7×10^9^/L; lymphocytes, 1.2×10^9^/L; hemoglobin, 9.7 g/dL (mean corpuscular volume: 92.9 fL); and hematocrit, 28.6%. His C-reactive protein (CRP) was 9.58 mg/dL, N-terminal-pro hormone B-type natriuretic peptide (NT-proBNP) was 2,512 pg/mL (normal value: <55 pg/mL), plasmin-alpha-2 plasmin inhibitor complex (PIC) was 15.9 μg/mL (normal value: <0.8 μg/mL), and thrombin-antithrombin complex (TAT) was 45.9 ng/mL (normal value: <3 ng/mL). The scoring system for DIC established by the Japanese Society on Thrombosis and Hemostasis was 7 points (threshold for DIC diagnosis is 6 points or more) [10]. Significantly elevated TAT, PIC, FDP, and DD suggest that the classification of DIC was enhanced fibrinolytic type [11]. Protein C activity was slightly decreased at 60% (reference range: 64%-146%), and free protein S antigen was within normal limits at 71% (reference range: 50%-131%). Antiphospholipid antibodies were negative. Lung tumor markers were almost normal: carcinoembryonic antigen (CEA), 4 ng/mL (normal value: <5 ng/mL); pro-gastrin-releasing peptide (ProGRP), 82.7 pg/mL (normal value: <81 pg/mL); and cytokeratin 19 fragments (CYFRA 21-1), 1.9 ng/mL (normal value: <3.5 ng/mL). Contrast-enhanced CT scan revealed pulmonary embolism in the right pulmonary arteries, ground glass opacity in the right lung base, left pleural effusion, and intramuscular hemorrhage-like swelling in the left back (Figure 1). CT and ultrasound detected no deep vein thrombosis.

**Figure 3:**
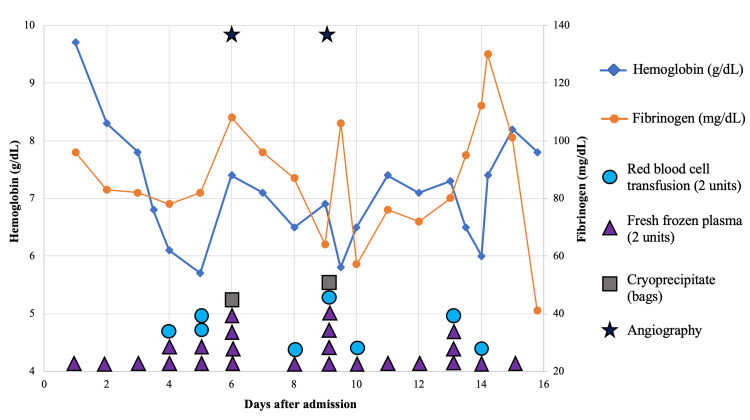
Clinical course We used a large amount of blood products for hypofibrinogenemia and multiple bleeding episodes.

On day 4 of admission, the anemia of the patient worsened, and contrast-enhanced CT revealed bleeding in the intramuscular artery near the left scapula. We performed angiography and detected bleeding from the branches of the left lateral thoracic artery. We successfully stopped the bleeding by embolization. On day 5, however, his hemoglobin level dropped again, and contrast-enhanced CT scan showed extravasation from the left intercostal artery. We moved on to angiography again and found extravasation from the sixth left intercostal artery and occluded it. On day 9, anemia deteriorated again, and contrast-enhanced CT detected intramuscular hemorrhage in the left chest wall and a pseudoaneurysm with hematoma and extravasation in the right femoral artery, where the arterial sheath was inserted in the previous angiography. We performed endovascular embolization and surgical aneurysm repair at the same time. On day 13, we performed bronchoscopy and found no mass lesion. We collected bronchoalveolar lavage fluid from the right B2 and left B3 bronchi. We were not able to obtain a histology specimen because of the coagulation abnormality. The cytology was negative (class II). After bronchoscopy, the hemoglobin level decreased; the cause was bleeding from the right femoral artery aneurysm. Cardiovascular surgeons performed surgical repair and stopped the bleeding. On day 15, the patient's respiratory and circulatory conditions worsened. Rapid ultrasound for shock and hypotension showed right ventricular dilatation and suggested worsening of pulmonary embolism as a likely cause. However, intervention for pulmonary embolism was impossible because we were unable to control the multiple bleeding sites. Administration of anticoagulants or antifibrinolytic agents was difficult due to the potential harm of controlling hemorrhagic shock and pulmonary embolism. He died that night. We used massive transfusion throughout the course because of persistently low fibrinogen levels, resulting in multiple bleeding events: 48 units of fresh frozen plasma, two bags of cryoprecipitate, and 16 units of red blood cell concentrate (Figure 3).

We tried tranexamic acid, unfractionated heparin, and gabexate mesilate for a few days, but they did not work, and we were not able to control bleeding or embolism.

**Figure 4:**
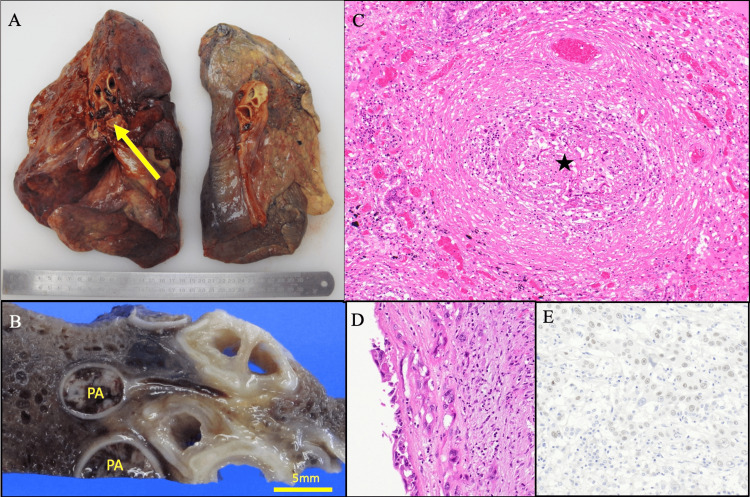
Autopsy findings A: Macroscopic finding of the lungs before formalin fixation. B: Macroscopic finding of the right lung after formalin fixation. A thrombus was confirmed in the right PA (yellow arrow). C: Microscopic finding (H&E, ×10): PA (★) is occluded by carcinoma and fibrosis. D: Microscopic finding (H&E, ×40): carcinoma showed creeping growth on the PA wall. E: Microscopic finding (immunohistochemistry): p40, which is an excellent marker for squamous cell carcinoma, was positive. PA: pulmonary artery, H&E: hematoxylin and eosin stain

We performed an autopsy to investigate the cause of death because we were not able to identify the underlying condition of DIC before his death. Autopsy revealed remarkable right lung swelling, massive right pulmonary embolism and tumor thrombosis, right lung squamous cell carcinoma with right pulmonary artery endovascular invasion, left lung adenocarcinoma, and left shoulder intramuscular hematoma. He had bilateral lung cancer, but the pathological findings were different: squamous cell carcinoma in the right lung and adenocarcinoma in the left lung. Right lung squamous cell carcinoma invaded into the right pulmonary artery along the endothelium (Figure 4).

However, no other malignancies, lymph nodes, or distant metastases were detected.

## Discussion

We experienced a case of severe chronic DIC caused by lung cancer and its pulmonary artery invasion. Imaging studies detected the lung nodules before the patient's death, but the sizes were small, and we thought they were not likely to be the cause of the refractory DIC. Also, fibrinogen was continuously very low, and we were not able to obtain biopsy samples to confirm the diagnosis. The treatment of DIC is the improvement of underlying conditions and supportive care [1,2]. However, in the presented case, making the diagnosis of underlying diseases was not possible, and we were not able to offer effective management.

DIC secondary to cancer often appears with an insidious and protracted clinical course [3]. This patient had a four-month history of bleeding tendency. Lung cancer and DIC had been active longer than that period.

In this case, DIC was very critical, and especially, bleeding was difficult to control because of the low fibrinogen level. Although an article reported that adenocarcinoma in lung cancer is more frequently the cause of DIC and thromboembolism [12], the invasion of squamous cell carcinoma along the endothelium and the resulting release of tissue factor are supposed to have a greater impact on our patient.

The endothelium plays an important role in the mechanism of DIC. In a normal physiological environment, the endothelium surface boosts the effect of endogenous anticoagulant proteins, antithrombin and protein C. Heparan sulfates in the glycocalyx enhance the inhibitory effect of antithrombin on thrombin. Endothelial protein C receptor and thrombomodulin show up on the stable endothelial cell surface, and they are necessary to activate protein C. However, endothelium damage increases vascular permeability and stimulates subendothelial activators (e.g., tissue factor and collagen). Abnormal control of tissue factor and thrombomodulin expression on the endothelial cells may induce increased thrombin formation, resulting in fibrinogen to fibrin conversion and stronger platelet activation. Anticoagulant proteins can also decline because of extravasation through the space between endothelia. In addition, endothelial activation or damage detaches glycocalyx and surface proteins from the endothelia by proteases, and those proteins circulate in the blood. The endothelium loses its anticoagulant potency [1]. Endothelial cells also provide urokinase-type plasminogen activator and tissue-type plasminogen activator and regulate plasminogen activator inhibitor type 1 (PAI-1) and fibrinolysis. Injured endothelium can lead to endothelial dysfunction and consequent insufficient release of plasminogen activators and higher levels of PAI-1. This may result in the overall antifibrinolytic state [3].

Although uncommon, squamous cell carcinoma invasion along the endothelium of the pulmonary artery has been reported [4-9]. Possible mechanisms are described in the literature. The first process that leads to tumor cell invasion into the lumen of blood and lymph vessels begins with chemotaxis toward the vessels. Tumor-associated macrophages (TAMs) play an essential role. They aggregate along blood vessels and tumor margins and secrete endothelial cell growth factor, which attracts the malignant cells to the vessels. Both mesenchymal and amoeboid migrating cells can invade blood vessels. Intravasation is completed either by the production of membrane extensions through spaces between endothelial walls, by passively going into vessels through preexisting entry sites, or by lining blood vessels and replacing endothelia, creating mosaic vessels [13,14].

For circulating tumor cells (CTCs) to be attached to endothelia, cohesive power between the CTC and the endothelium should be higher than the shear forces applied on the cell in the blood circulation. This endothelial adhesion process may start with a weak link, enabling the CTC to move on the vessel wall before a firmer affinity provokes a complete adhesion of cancer cells. CTCs are easier to stop at areas with slower bloodstream or weaker wall shear stress. Shear stress is not similar everywhere in the blood vessels in our body, and fluid dynamics in the main arteries can cause stronger shear stress [14]. An article described that strong shear stress can lead to weaker cell activity than cells in vitro, but white blood cells and malignant cells were less influenced by the temporarily increased shear stress in comparison to benign endothelial cells [15]. Higher shear stress may damage cells. On the other hand, lower shear stress may be beneficial for CTC survival [14]. In the presented case, tumor invasion was mainly observed in the right pulmonary artery, where blood pressure is significantly lower than systemic circulation.

Platelets can help cancer spread by sticking to CTCs and making clusters. Aggregation of CTCs and platelets may assist in the prevention of anoikis, reduce shear stress on tumor cells, prevent their detection by leukocytes, diminish natural killer cell activity, and increase the possibility of resting in an arrested state. Neutrophils may also promote CTC survival. Neutrophil extracellular traps are able to support linking to blood vessel walls and assist metastasis [14]. Another article reported clusters of tumor cells and leukocytes and revealed that plenty of myeloid cells manifested morphologies compatible with neutrophils. Unlike individual CTCs, cancer cells from CTC-neutrophil complexes enhance cell cycle-promoting genes and show more rapid spread of cancer cells in mice after intravenous injection [16]. In addition, the CTC-neutrophil complex was related to poorer prognosis in cancer patients [14]. Im et al. reported that platelet-fibrin clots surround cancer cells when tumor cells are separated from the primary tumor into the circulation [17]. These clots can preserve tumor cells from immunologic and physiological stresses in the blood vessels. Then, the tumor cell α3β1 integrin communicates with laminin-5 exposed on the basement membrane in the endothelium of the pulmonary vasculature [18]. This process facilitates the attachment of tumor cells to the endothelium of pulmonary vessels. Honn et al. suggested that platelet activation can provoke retraction of endothelial cells in the pulmonary blood vessels [19]. This retraction may increase the available area of the basement membrane and offer a surface for tumor cell settlement. Our patient's platelet was preserved, and the WBC count was increased, which can be protective for CTC survival.

## Conclusions

We reported the first case of catastrophic DIC secondary to squamous cell carcinoma of the lung and its intravascular invasion into the pulmonary artery along the endothelium. Tumor extension inside the pulmonary vasculature along the endothelium could damage endothelial cells, and this leads to the activation of multiple mechanisms for tumor spreading and uncontrollable DIC. Imaging studies, including contrast-enhanced CT, were not able to detect intravascular invasion of lung cancer. We have to consider the possibility of an intravascular tumor in patients with DIC of unknown cause.
